# Mid-term outcomes of trans-axillary versus thoracotomy approaches in alternative-access tavr: a retrospective multicenter study

**DOI:** 10.1007/s12928-026-01253-7

**Published:** 2026-02-28

**Authors:** Chiaki Aichi, Masahiro Inagaki, Junji Yanagisawa, Tetsuro Shimura, Masanori Yamamoto, Hideki Kitamura, Yutaka Koyama

**Affiliations:** 1 Department of Cardiovascular Surgery, Nagoya Heart Center, 1-1-14 Sunadabashi, Higashi-ku, Nagoya City, Aichi Prefecture 461-0045 Japan; 2 Department of Cardiovascular Surgery, Gifu Heart Center, Gifu, Japan; 3 Department of Cardiovascular Surgery, Toyohashi Heart Center, Toyohashi, Japan; 4 Department of Cardiology, Gifu Heart Center, Gifu, Japan; 5 Department of Cardiology, Toyohashi Heart Center, Toyohashi, Japan

**Keywords:** Transcatheter aortic valve replacement, Alternative approaches, Mid-term outcomes, Thoracotomy

## Abstract

Transcatheter aortic valve replacement (TAVR) has several alternative access routes when transfemoral access is unsuitable. This study compared perioperative and mid-term outcomes of transaxillary (TAx) TAVR with those of transapical (TA) and direct aortic (DA) approaches. Among 2,185 patients who underwent TAVR at our three centers between April 2015 and April 2024, 198 non-transfemoral cases were retrospectively analyzed. TAx-TAVR was classified as a non-thoracotomy approach, whereas TA/DA-TAVR were classified as thoracotomy approaches. Perioperative and mid-term outcomes were compared between the two groups. Of the 198 patients, 97 underwent the non-thoracotomy approach and 101 underwent thoracotomy (TA, *n* = 64; DA, *n* = 37). Most baseline characteristics were comparable, although dialysis-dependent patients were more common in the non-thoracotomy group. Operative time, transfusion requirement, and hospital stay were significantly greater in the thoracotomy group. Access-related complications included four events in the thoracotomy group (aortic injury or apical rupture) and five events in the non-thoracotomy group (aortic dissection or intimal injury). Postoperative ischemic stroke tended to occur more frequently in the non-thoracotomy group, though not significantly. One-year survival was significantly higher in the non-thoracotomy group, whereas mid-term mortality did not differ between groups. TAx-TAVR was associated with lower perioperative invasiveness and superior one-year survival compared with TA/DA-TAVR. However, the trend toward increased postoperative stroke underscores the importance of careful patient selection and thorough preoperative vascular assessment.

**Graphical Abstract:**

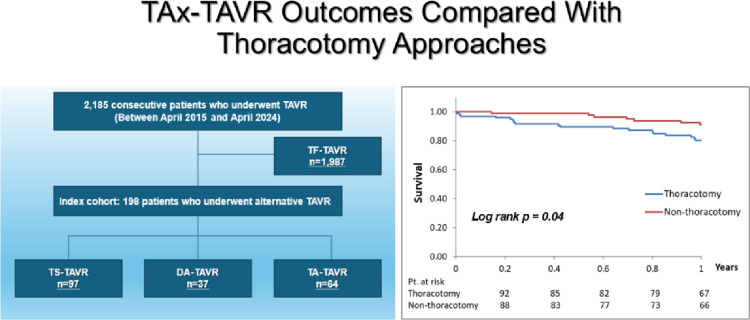

**Supplementary Information:**

The online version contains supplementary material available at 10.1007/s12928-026-01253-7.

## Introduction

Since the first report in 2002 [1], transcatheter aortic valve replacement (TAVR) has primarily been performed in patients at high surgical risk. The safety of transfemoral (TF) TAVR, the most commonly used access route, has been well established [2]. However, TF-TAVR is not feasible in approximately 10–15% of patients [3], necessitating alternative access routes such as transaxillary (TAx), direct aortic (DA), transapical (TA), and transcarotid (TC) approaches. Although various alternative access routes have been proposed, no clear evidence indicates the superiority of one approach over another, and selection criteria vary widely among heart teams. At our institution, TAx-TAVR is adopted as the first-line alternative access; however, its clinical outcomes remain insufficiently characterized. In this study, we compared the perioperative and mid-term outcomes of TAx-TAVR (non-thoracotomy approach) with those of TA- and DA-TAVR (thoracotomy approaches).

## Methods

### Study population

**Fig. 1:**
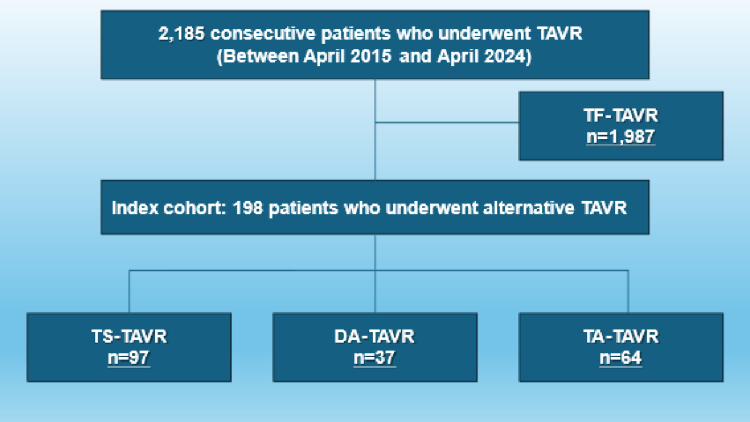
Between April 2015 and April 2024, 2,185 patients underwent TAVR at our centers. Of these, 198 patients who underwent TAVR using non-TF approaches were included in this retrospective study, with TS-TAVR grouped as a non-thoracotomy approach, and DA-TAVR and TA-TAVR grouped as thoracotomy approaches

Among 2,185 patients who underwent TAVR at three participating institutions between April 2015 and April 2024, a total of 198 patients who underwent non-transfemoral (non-TF) TAVR were included in this study (Fig. 1). This study was approved by the institutional review board (IRB number: 2024031), and the requirement for individual informed consent was waived. Clinical data were retrospectively extracted from electronic medical records.

### Study design and endpoints

The primary endpoint of this study was all-cause mortality. Secondary endpoints were adverse events occurring within 30 days after the procedure. Bleeding, stroke, vascular complications, and other adverse events were defined according to the Valve Academic Research Consortium-3 (VARC-3) criteria.

### Surgical technique

TAx-TAVR was performed under local anesthesia. The left subclavian artery was the preferred access route; the right subclavian artery was used only when the left internal thoracic artery had been harvested for coronary artery bypass grafting or when the left subclavian artery was anatomically small, because the angle toward the aortic annulus is generally unfavorable from the right side.

Thoracotomy approaches were performed under general anesthesia. In DA-TAVR, the ascending aorta was exposed via an upper hemi-sternotomy or right mini-thoracotomy. TA-TAVR was performed via a left anterolateral mini-thoracotomy with exposure of the left ventricular apex, without single-lung ventilation.

### Statistical analysis

Continuous variables were expressed as mean ± standard deviation or median (interquartile range), and categorical variables as frequencies and percentages. Statistical analyses were performed using EZR (Easy R; Saitama Medical Center, Jichi Medical University). Group comparisons of continuous variables were conducted using the t-test or the Mann–Whitney U test depending on normality, whereas categorical variables were compared using the chi-square test. Survival curves were generated using the Kaplan–Meier method, and differences between groups were assessed using the log-rank test. Multivariable analysis was performed using logistic regression or the Cox proportional hazards model, depending on the nature of each outcome. Covariates included in the multivariable models were selected based on clinical relevance, such as age, sex, and access approach, as well as variables that showed statistical significance in univariable analyses. A P value of < 0.05 was considered statistically significant. In addition, sensitivity analyses excluding dialysis-dependent patients were performed to account for the potential impact of maintenance dialysis on prognosis.

## Results

### Baseline characteristics

In the overall cohort, 97 patients underwent the non-thoracotomy approach and 101 patients underwent the thoracotomy approach. There were no significant differences between the two groups in age, sex, Clinical Frailty Scale, or Society of Thoracic Surgeons score.　However, dialysis-dependent patients were significantly more frequent in the non-thoracotomy group, and eGFR was significantly lower in this group (Table 1).

**Table 1:** Baseline characteristics (*N* = 198)

	Thoracotomy (*N* = 101)	Non- Thoracotomy (*N* = 97)	*P* Value
Age (years)	**83.3 ± 5.7**	**84.5 ± 5.3**	**0.113**
Male	**42 (42%)**	**54 (56%)**	**0.064**
Body Mass Index (kg/m²)	**21.3 ± 3.0**	**20.7 ± 3.2**	**0.183**
Body Surface Area (m²)	**1.41 ± 0.2**	**1.45 ± 0.2**	**0.132**
Hypertension	**93 (92%)**	**86 (89%)**	**0.603**
Diabetes Mellitus	**38 (38%)**	**29 (30%)**	**0.294**
Peripheral vascular disease	**45 (45%)**	**40 (41%)**	**0.668**
Hemoglobin (g/dL)	**11.3 ± 1.7**	**11.6 ± 1.6**	**0.679**
eGFR (mL/min/1.73 m²)	**41.4 ± 20**	**34.2 ± 23**	**0.021**
Dialysis	**13 (13%)**	**27 (28%)**	**0.013**
COPD	**13 (13%)**	**16 (16%)**	**0.548**
Albumin (g/dL)	**3.6 ± 0.6**	**3.6 ± 0.5**	**0.837**
NT-proBNP (pg/mL)	**7978 ± 11,876**	**10,611 ± 13,373**	**0.162**
History of stroke	**9 (%)**	**15 (%)**	**0.193**
Atrial fibrillation	**15 (15%)**	**17 (18%)**	**0.700**
Previous PCI	**28 (28%)**	**38 (39%)**	**0.132**
Previous cardiac surgery	**14 (14%)**	**11 (11%)**	**0.671**
Previous CABG	**12 (12%)**	**6 (6%)**	**0.217**
NYHA ≥ Ⅲ,Ⅳ	**30 (30%)**	**33 (34%)**	**0.576**
STS	**10.16 ± 6.32**	**11.56 ± 7.1**	**0.145**
CFS ≥ 4	**71 (70%)**	**70 (70%)**	**0.875**
Emergent Procedure	**5 (5.0%)**	**5 (5.1%)**	**1.000**
LVEF (%)	**58 ± 14**	**57 ± 15**	**0.908**
Mean PG (mmHg)	**46 ± 15**	**44 ± 16**	**0.308**
AVA (cm²)	**0.65 ± 0.2**	**0.68 ± 0.2**	**0.290**
MR ≥ moderate	**13 (13%)**	**23 (23%)**	**0.065**
TR ≥ moderate	**11 (11%)**	**20 (21%)**	**0.078**

eGFR = estimated glomerular filtration rate; COPD = chronic obstructive pulmonary disease; STS = Society of Thoracic Surgeons; CFS = Clinical Frailty Scale; PCI = percutaneous coronary intervention; NYHA = New York Heart Association, LVEF = left ventricular ejection fraction; PG = pressure gradient; AVA = aortic valve area; MR = mitral regurgitation; TR = tricuspid regurgitation

### Operative outcomes

Operative time was significantly longer in the thoracotomy group (121 min vs. 78 min, *p* < 0.001). There were also differences in device selection: self-expandable valves were more commonly used in the non-thoracotomy group, whereas balloon-expandable valves were significantly more frequent in the thoracotomy group (*p* < 0.001). In addition, the distribution of transcatheter heart valve types and sizes used in this study is shown in Supplementary Figure S1. The thoracotomy group experienced significantly higher rates of both life-threatening bleeding and overall bleeding events and required blood transfusion more frequently (25 vs. 13, *p* = 0.02). In addition, postoperative hospital stay was significantly longer (8.5 days vs. 5 days, *p* = 0.003). Approach-related complications included aortic injury (*n* = 2), apical rupture (*n* = 1), and left anterior descending artery compression due to apical hematoma (*n* = 1) in the thoracotomy group. In the non-thoracotomy group, aortic dissection (*n* = 2) and intimal injury requiring repair (*n* = 3) were observed. Ischemic stroke occurred in 5 patients in the non-thoracotomy group and in 1 patient in the thoracotomy group, with no significant difference (*p* = 0.11).

In-hospital mortality occurred only in the thoracotomy group (5 cases), with causes including pneumonia (*n* = 3), acute aortic dissection (*n* = 1), and aortic root rupture (*n* = 1), although the difference was not statistically significant (*p* = 0.06) (Table 2).

**Table 2:** Operative outcomes (*N* = 198)

	Thoracotomy (*N* = 101)	Non-Thoracotomy (*N* = 97)	*P* Value
Operative time (min)	**121 ± 87**	**78 ± 18**	**< 0.001**
Self-expandable	**11 (11%)**	**45 (46%)**	**< 0.001**
Balloon-expandable	**89 (88%)**	**51 (53%)**	**< 0.001**
BAV	**42 (42%)**	**29 (30%)**	**0.103**
Post-dilatation	**10 (9.9%)**	**5 (5.2%)**	**0.284**
Valve-in-valve	**0**	**4 (4.1%)**	**0.056**
Contrast agent (ml)	**79 ± 56**	**67 ± 41**	**0.089**
AR ≥ moderate	**0**	**4 (4.1%)**	**0.444**
Mean PG	**10.6 ± 4.7**	**9.7 ± 4.6**	**0.345**
EOA	**1.64 ± 0.54**	**1.76 ± 0.48**	**0.165**
Postoperative hospital stay	**8.5 (7–14)**	**5 (4–7)**	**0.003**
Access complications	**4 (4.0%)**	**5 (5.1%)**	**0.744**
Coronary complications	**1 (1.0%)**	**1 (1.0%)**	**1.000**
Aortic root rupture	**2 (2.0%)**	**0**	**0.498**
Bleeding	**28 (28%)**	**11 (11%)**	**0.007**
Life-threatening bleeding	**6 (5.9%)**	**0**	**0.029**
Major bleeding	**13 (13%)**	**8 (8.2%)**	**0.29**
Minor bleeding	**9 (8.9%)**	**3 (3.1%)**	**0.09**
Blood transfusion	**34 (34%)**	**18 (19%)**	**0.002**
V-A ECMO	**4 (4.0%)**	**0**	**0.057**
New-onset AF	**6 (6.0%)**	**2 (2.1%)**	**0.057**
Ischemic stroke	**1 (1.0%)**	**5 (7.2%)**	**0.113**
Disabling ischemic stroke	**1 (1.0%)**	**4 (4.1%)**	**0.37**
Non-disabling ischemic stroke	**0**	**1 (1.0%)**	**1.00**
Hemorrhagic stroke	**0**	**0**	**N/A**
Acute kidney injury	**5 (5.0%)**	**3 (3.1%)**	**0.537**
Pacemaker implantation	**4 (4.0%)**	**7 (7.2%)**	**0.537**
In-hospital death	**5 (5.0%)**	**0**	**0.060**

BAV = Balloon Aortic Valvuloplasty; AR = aortic valve regurgitation; PG = pressure gradient; EOA = effective orifice area; V-A ECMO = veno-arterial extracorporeal membrane oxygenation; AF = atrial fibrillation

**Table 3:**
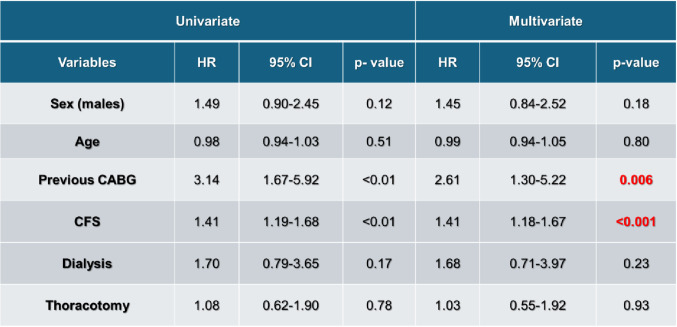
Predictors of all-cause mortality

CABG = coronary artery bypass grafting; CFS = clinical frailty scale; CI = confidence interval; HR = hazard ratio

### Survival probability

**Fig. 2:**
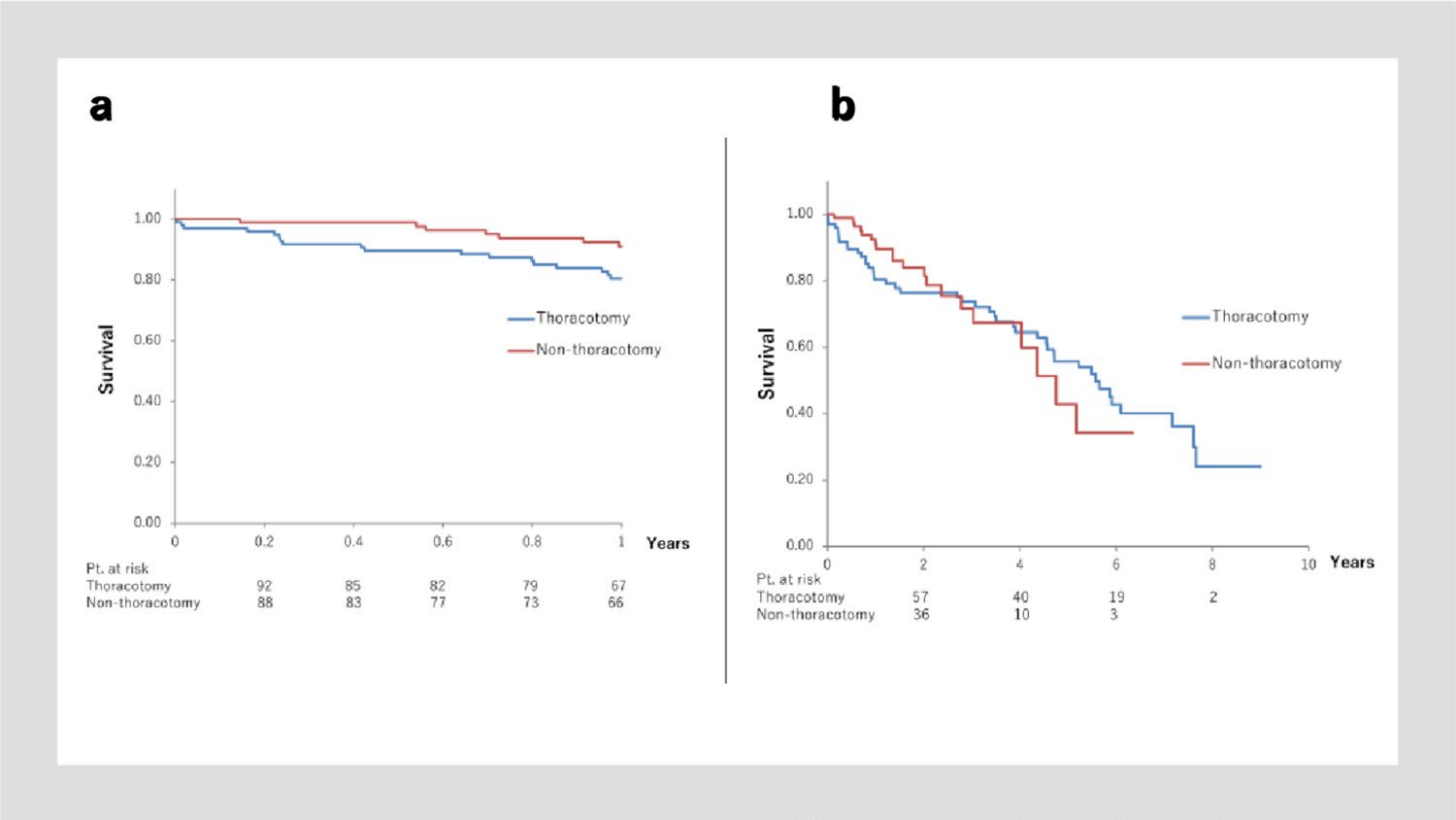
One-year survival was significantly higher in the non-thoracotomy group (a; 91.1% vs. 80.4%, *p* = 0.04). In contrast, mid-term mortality did not differ significantly between the two groups (b; median survival 5.58 years vs. 4.76 years, *p* = 0.78)

One-year survival was significantly higher in the non-thoracotomy group (91.1% vs. 80.4%, *p* = 0.04). In contrast, mid-term mortality did not differ significantly between the two groups (median survival 5.58 years vs. 4.76 years, *p* = 0.78) (Fig. 2). In the multivariable Cox proportional hazards analysis for all-cause mortality, access route was not an independent predictor. Instead, preoperative Clinical Frailty Scale (HR 1.41, 95% CI 1.18–1.67, *p* < 0.01) and history of coronary artery bypass grafting (HR 2.61, 95% CI 1.30–5.22, *p* = 0.01) were identified as independent predictors of poor prognosis (Table 3). In sensitivity analyses excluding dialysis-dependent patients, the trend in survival outcomes was generally consistent with that observed in the overall analysis. These results are presented in Supplementary Figure S2.

## Discussion

In this study, we compared the perioperative and mid-term outcomes of TAx-TAVR (non-thoracotomy approach) with those of TA and DA-TAVR (thoracotomy approaches) performed as alternative access routes. The thoracotomy group showed a higher tendency toward in-hospital mortality. Most deaths were attributed to pneumonia, which may be associated with impaired respiratory function due to thoracotomy and general anesthesia, as well as delayed postoperative recovery. This difference in early mortality was reflected in the significantly lower 1-year survival rate in the thoracotomy group, underscoring the advantage of the TAx-TAVR. These results suggest that TAx-TAVR is a reasonable and less invasive alternative access option for patients in whom TF-TAVR is not feasible. Consistent with previous reports, TAx-TAVR in our cohort was associated with shorter operative time and reduced length of hospital stay, indicating lower procedural burden for patients [4–6].

On the other hand, although not statistically significant, postoperative ischemic stroke tended to occur more frequently in the TAx-TAVR group. This finding is consistent with previous studies [7, 8]. One potential explanation is that delivery systems introduced via the subclavian artery may come into contact with atherosclerotic plaques in the aortic arch, increasing the risk of embolic debris dislodgement. Vertebral artery occlusion may also lead to posterior circulation ischemic stroke [9]. In addition, the more frequent use of self-expandable valves in TAx-TAVR may increase embolic risk due to the characteristics of the deployment process [10]. In the present study, ischemic stroke occurred in one patient who underwent DA-TAVR and in five patients who underwent TAx-TAVR. In the DA-TAVR case, severe bilateral carotid artery stenosis had been identified preoperatively, which was considered a possible cause of the ischemic stroke. In contrast, among the TAx-TAVR cases, two patients had atherosclerotic plaques in the aortic arch, one patient had protruding calcification into the lumen of the ascending aorta, one patient developed postoperative Stanford type A aortic dissection, and one patient had severe calcification of the aortic valve.

These findings suggest that the mechanisms underlying ischemic stroke after TAx-TAVR are not uniform but rather multifactorial, involving aortic arch atheroma, extensive calcification of the ascending aorta and annulus, and postoperative complications, with different contributing factors in individual cases. Indeed, no clear causal relationship was observed between stroke occurrence and dominance of the access-side vertebral artery in this study. Therefore, when considering TAx-TAVR, comprehensive vascular assessment is essential, including not only laterality of vertebral artery dominance but also evaluation of aortic arch atheroma and calcification of the ascending aorta and aortic valve. Moreover, in the present study, three cases of intimal injury requiring repair and two cases of aortic dissection occurred in the TAx-TAVR, raising concern that the mismatch between vascular diameter and the delivery system may increase vascular stress. In particular, for patients with a minimal subclavian artery diameter < 5.5 mm, alternative access routes may need to be considered.

**Fig. 3:**
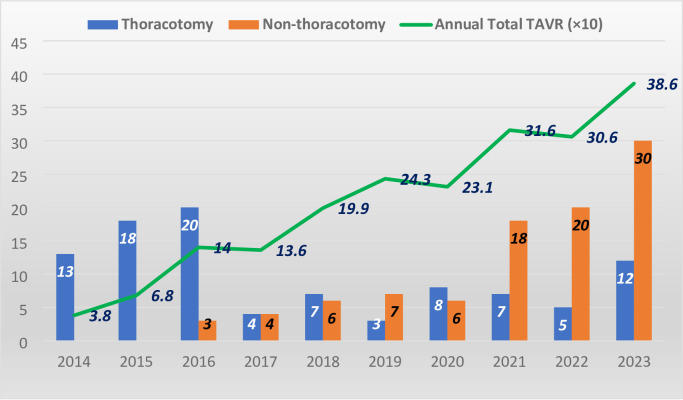
Number of cases in which the thoracotomy and non-thoracotomy approaches were used each year. In 2013, the Sapien XT (Edwards Lifesciences Ltd, Irvine, CA, USA) received regulatory approval for TF and TA-TAVR. Subsequently, in 2015, the CoreValve (Medtronic Inc., MN, USA) was approved for TF, DA, and TS-TAVR. Our institution performed its first TS-TAVR in 2017. Additionally, since SAPIEN 3 (Edwards Lifesciences Ltd, Irvine, CA, USA) received regulatory approval for TS-TAVR and use in dialysis patients in 2021, the overall number of TAVR procedures, as well as in the absolute number and proportion of TS-TAVR, have shown an increasing trend

In Japan, CoreValve (Medtronic Inc., MN, USA) was approved for TAx-TAVR in 2015, and our institution performed its first TAx-TAVR in 2016. Subsequently, with the approval of SAPIEN 3 (Edwards Lifesciences Ltd., Irvine, CA, USA) for TAx-TAVR and for use in dialysis patients in 2021, both the absolute number and proportion of TAx-TAVR procedures have shown a clear increasing trend (Fig. 3). This is likely due to the high prevalence of severe peripheral arterial calcification in dialysis patients, making TF-TAVR anatomically unsuitable in many cases. Given the relatively high proportion of dialysis patients in Japan, the demand for alternative access routes is expected to continue increasing nationwide.

Among alternative access options, the transcarotid (TC) approach has recently gained attention. TC-TAVR offers a favorable trajectory to the aortic annulus and may be associated with a lower risk of embolic events [11, 12], and accumulating evidence regarding long-term outcomes and cerebrovascular events is anticipated.

### Limitations

This study has several limitations. First, it was a retrospective observational study, and the choice of access route depended on the operator’s judgment and institutional policy. Therefore, selection bias and residual confounding cannot be fully excluded. Second, a larger proportion of thoracotomy cases were performed during the early study period, and improvements in device technology and procedural techniques over time may have influenced perioperative outcomes and prognosis. Third, the overall sample size was relatively limited, and for low-frequency events such as in-hospital mortality and ischemic stroke, the statistical power may have been insufficient to detect significant differences. Third, to enable a two-group comparison, TA and DA approaches were combined into a single thoracotomy group in this study. However, there may be inherent heterogeneity between the TA and DA approaches in terms of procedural characteristics and complication profiles, which represents one of the limitations of this study.

## Conclusion

The present study demonstrated that TAx-TAVR is a reasonable secondary access option to replace TF-TAVR in terms of safety and minimal invasiveness, while also highlighting the potential risk of ischemic stroke. Careful pre-procedural evaluation—including assessment of aortic arch atheroma and vertebral artery blood flow—is essential, along with appropriate device selection and meticulous handling tailored to the vessel diameter.

## Electronic Supplementary Material

Below is the link to the electronic supplementary material.

Supplementary Material 1

## Data Availability

The Data is available and will be provided at the editor’s request.
