# Supplementary material for: Mid-term outcomes of trans-axillary versus thoracotomy approaches in alternative-access tavr: a retrospective multicenter study
Source: Cardiovasc Interv Ther. 2026 Feb 28;41(3):717–23. doi: 10.1007/s12928-026-01253-7 (PMC13279667; doi:10.1007/s12928-026-01253-7)
Supplement: Supplementary file 1 — Supplementary Material 1 [file 12928_2026_1253_MOESM1_ESM.docx]

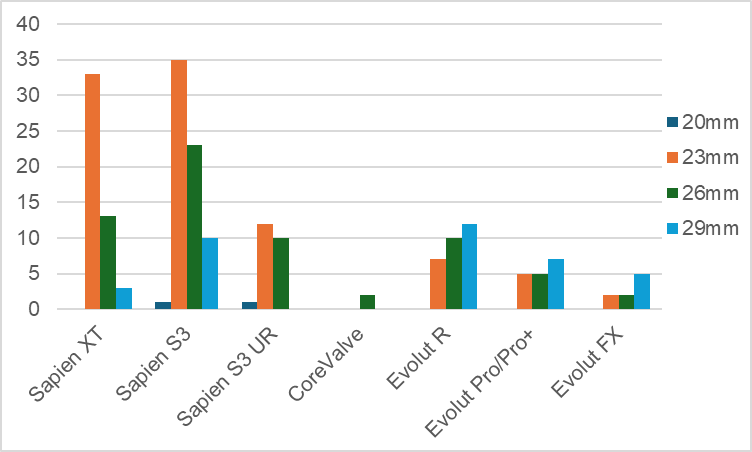


Figure S1 The distribution of valve types and sizes is shown graphically. In the SAPIEN series (Edwards Lifesciences, USA), 23-mm valves were most frequently used, whereas in the Evolut series (Medtronic, USA), there was a tendency toward more frequent use of 29-mm valves.


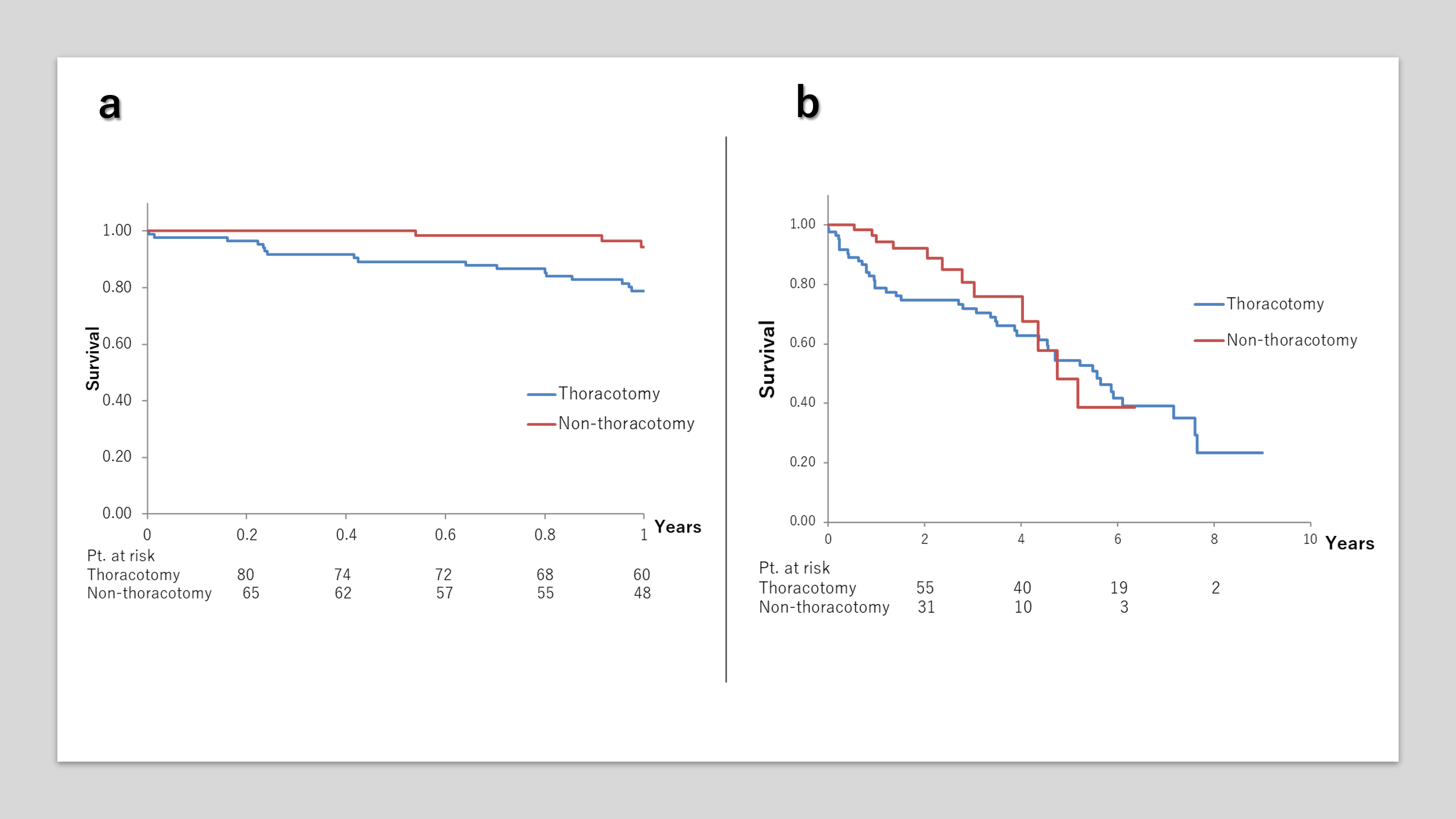


Figure S2 Even in sensitivity analyses excluding dialysis-dependent patients, one-year survival remained significantly higher in the non-thoracotomy group (a; 78.8% vs. 94.4%, p = 0.008), whereas no significant difference was observed between the two groups in mid-term survival (b; median survival 5.12 vs. 4.67 years, p = 0.25).
